# Biparietal Osteodystrophy Visualized on a Dermatologist’s Scalp Examination

**DOI:** 10.7759/cureus.74879

**Published:** 2024-11-30

**Authors:** Kritin K Verma, Tramondranique Hawkins, Ryan Wealther, Daniel P Friedmann, Michelle Tarbox

**Affiliations:** 1 Medicine, Texas Tech University Health Sciences Center, Lubbock, USA; 2 Dermatology, Texas Tech University Health Sciences Center, Lubbock, USA; 3 Westlake Dermatology Clinical Research Center, Westlake Dermatology & Cosmetic Surgery, Austin, USA

**Keywords:** biparietal osteodystrophy, dermatology, examination, osteoporosis, scalp deformity, skin exam

## Abstract

Biparietal osteodystrophy, also known as biparietal thinning, is an uncommon illness that causes symmetrical thinning of the parietal bones. It primarily affects women in their sixth and seventh decades of life. We present a case of a 73-year-old female with osteoporosis and osteoarthritis who visited a dermatology clinic with a bulging mass around her vertex scalp, accompanied by episodes of dizziness and blurred vision. The patient had a history of head trauma, but the skull abnormality was observed prior to these episodes. Laboratory testing was unremarkable except for high vitamin D levels. Imaging studies, including CT and MRI, revealed bilateral parietal bone loss without cerebral anomalies. The patient was referred to neurology for further evaluation. This patient’s presentation emphasizes the significance of including biparietal osteodystrophy in the differential diagnosis when patients report scalp abnormalities. Dermatologists should be aware of this condition and its potential links to osteoporosis and other systemic diseases.

## Introduction

Biparietal osteodystrophy, also known as biparietal thinning, is a rare condition characterized by symmetrical thinning of the parietal bones [1]. This illness typically manifests in the sixth and seventh decades of life and is more prevalent in women [2]. The etiology of biparietal osteodystrophy is unknown, although suspected associations include osteoporosis, calvarial trauma, inflammation, long-term steroid usage, malignancy, diabetes, hyperparathyroidism, granulomatous illness, and Gorham-Stout syndrome [2-5].

Biparietal osteodystrophy may manifest as a scalp deformity, often with pain on presentation [1]. This condition may be discovered incidentally in the dermatology clinic during standard scalp exams. However, imaging such as skull radiography and computed tomography (CT) scans, remain the golden standard for diagnosis as they reveal the typical thinning of the parietal bones [3]. Biparietal osteodystrophy is rarely mentioned in dermatology literature, however, physicians may encounter patients complaining of skull deformity, dizziness, or blurred vision [3,4]. We describe a case of a 73-year-old female with biparietal osteodystrophy who presented with scalp deformities and other symptoms.

## Case presentation

**Figure 1:**
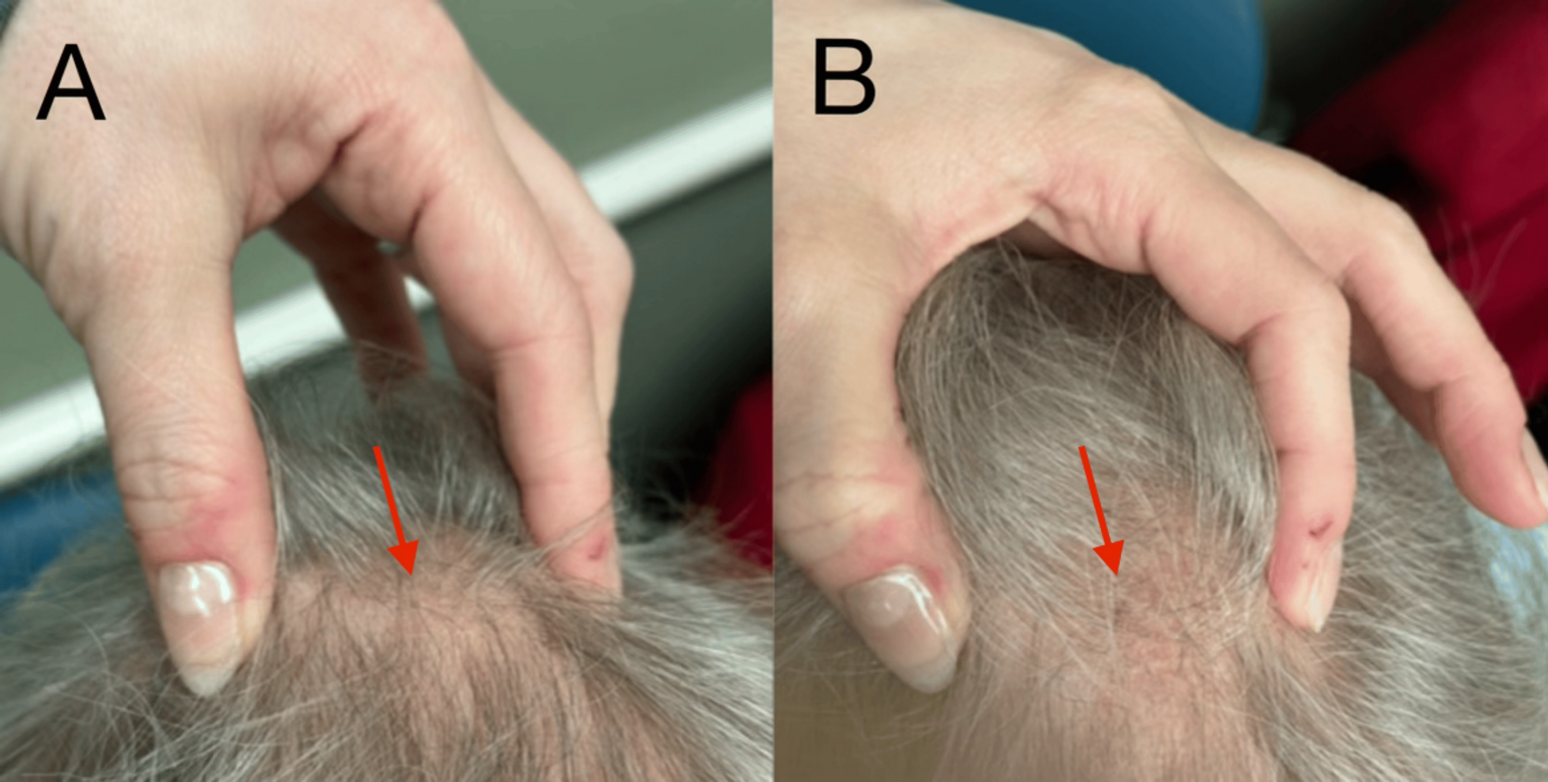
Biparietal osteodystrophy visualized on a scalp exam. Slightly horizontal (A) and vertical (B) views of the bulge palpated in the patient’s calvarium.

A 73-year-old female with a past medical history of osteoporosis and osteoarthritis presented to a dermatology clinic for a routine skin examination. Upon scalp exam, a bulging of the patient's cranium was noted (Figure 1A, 1B).

The patient noticed this on herself and reported dizziness and impaired vision. She had a history of head trauma in 2018 and 2021, although the malformation existed before the onset of these episodes. Due to an extensive history of vitamin D deficiency, the patient reported taking access amounts of Vitamin D supplements. Laboratory results at the time of the presentation are reported in Table 1.

**Table 1:** Initial laboratory values. eGFR: Estimated Glomerular Filtration Rate; Cr: Creatinine; BUN: Blood Urea Nitrogen.

Parameter	Patient's Results	Reference Range
25-hydroxy Vitamin D	120 ng/mL	20-40 ng/mL
eGFR	70.51 mL/min	90-120 mL/min
Cr	0.8 mg/dL	0.5-1.2 mg/dL
BUN	13 mg/dL	7-20 mg/dL
Alkaline Phosphatase	78 U/L	45-115 U/L
Calcium	9.7 mg/dL	8.5-10.2 mg/dL

**Figure 2:**
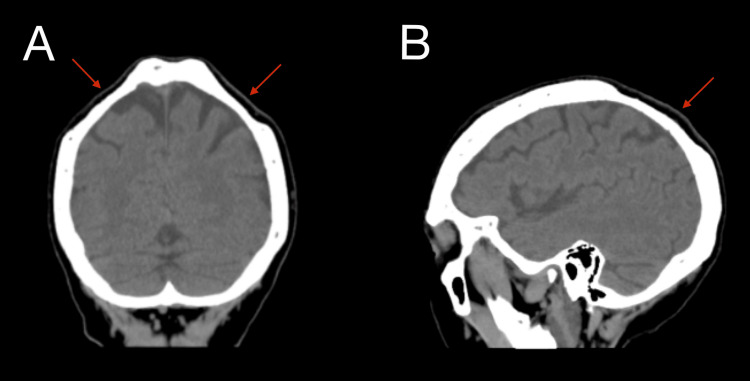
Biparietal osteodystrophy on CT imaging. Coronal (A) and sagittal (B) views demonstrate bilateral thinning of the parietal bone. CT: computed tomography

Computed tomography (CT) head imaging without contrast was ordered to identify other cranial abnormalities not visible superficially, which showed bilateral parietal bone loss (Figure 2A, 2B).

**Figure 3:**
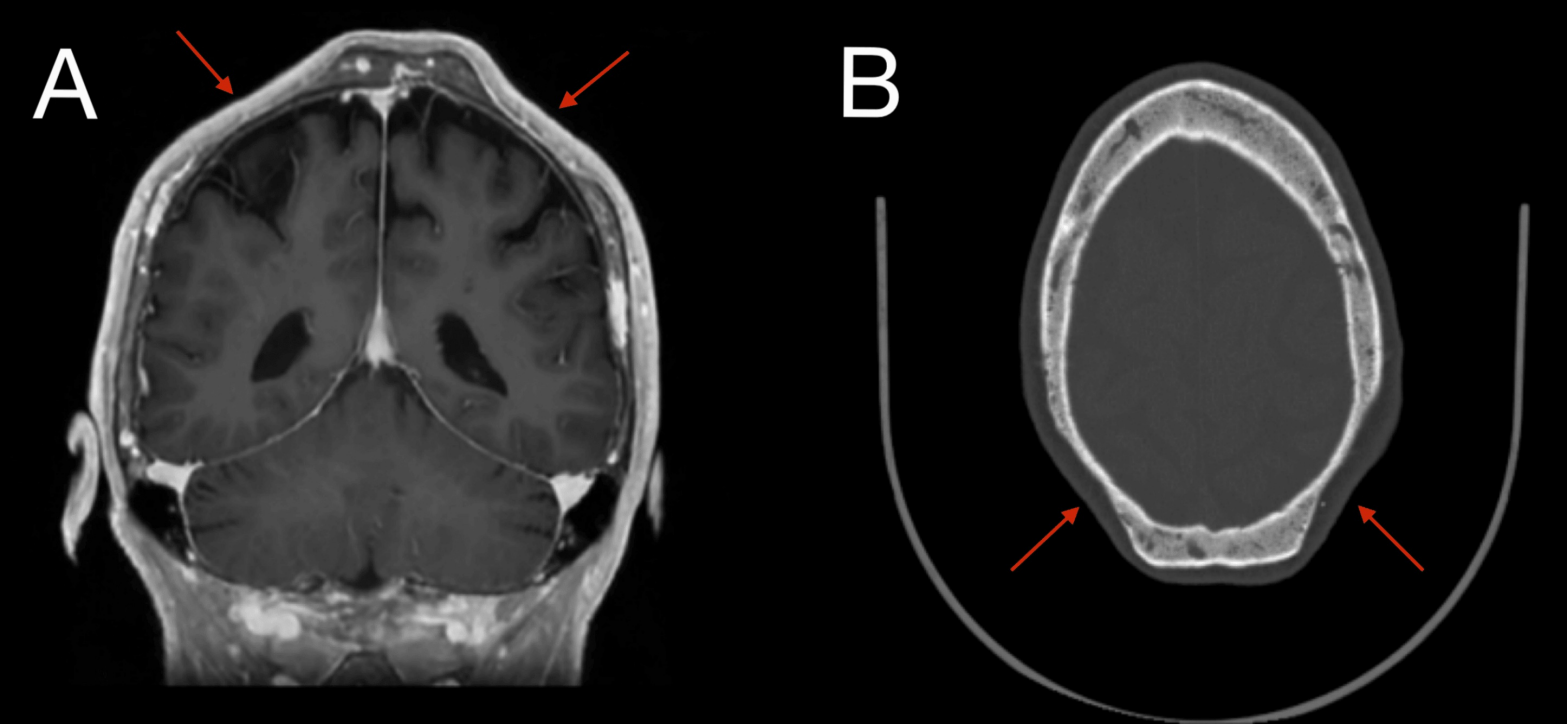
Biparietal osteodystrophy on MRI imaging. Coronal (A) and axial (B) views show bilateral thinning of the parietal bone and no underlying intracranial abnormalities. MRI: magnetic resonance imaging

Follow-up magnetic resonance imaging (MRI), with and without contrast, revealed no intracranial abnormalities (Figure 3A, 3B). The patient was referred to neurology for further evaluation.

## Discussion

Most cases of biparietal osteodystrophy do not cause neurological symptoms, although dizziness may present in rare occurrences [6], as demonstrated in this case. Biparietal osteodystrophy is rarely mentioned in the dermatology literature, however, dermatologists may encounter patients complaining of skull deformity or uncover it during scalp examinations [2,3]. In this case, there were divots bilaterally on the patient's head, which raised the suspicion of osteodystrophy.

The patient in this case was already being treated for osteoporosis, and a follow-up MRI indicated she was stable. When patients complain of any deformity during a scalp examination, osteodystrophy should be included in the differential diagnosis. Biparietal osteodystrophy can be identified by visible abnormalities on the head, such as bulging or thinning of the parietal bones, and may be mistaken for other illnesses [3]. Other conditions to consider in the differential diagnoses include illnesses that present with non-traumatic skull deformities, such as Paget's disease of bone, hyperparathyroidism, and other metabolic bone abnormalities [7,8]. These disorders may present with similar radiologic findings, hence, it is important to conduct thorough clinical and imaging assessments to distinguish them [7,8].

If patients present with divots on their scalp, osteodystrophy or underlying bone pathology should be considered. Dermatologists should be aware of the relationship between biparietal osteodystrophy and osteoporosis, in addition to secondary influences such as inflammation, long-term steroid use, and other systemic disorders [2,3]. Early detection and timely referral for imaging and specialist evaluation are essential in managing this rare illness.

## Conclusions

Biparietal osteodystrophy may be visualized upon scalp examinations by dermatologists. This case emphasizes the need to identify skull deformities and related symptoms, such as dizziness and visual impairment, which can signal underlying bone pathology. While normally asymptomatic, this illness can occasionally cause neurological symptoms. Dermatologists should be knowledgeable about biparietal osteodystrophy, its potential associations with osteoporosis and other systemic disorders, and its differential diagnoses. Early identification, appropriate imaging, and quick referral to experts are critical for the successful treatment regime of this unique condition. Dermatologists can help detect and treat patients with biparietal osteodystrophy by remaining vigilant during scalp inspections for indentations or irregular divots on a patient's head.

## References

[1] Sanati-Mehrizy P, Graziano FD, Naidich T, Taub PJ (2020). Characterization of bilateral parietal thinning. J Craniofac Surg.

[2] Watson L, Hovav O, Phua Y (2021). Focal idiopathic calvarial thinning: a condition of uncertain prevalence and significance. J Craniofac Surg.

[3] Yılmaz MB, Egemen E, Ozbakır B, Tekiner A (2013). Epidural hematoma after minor trauma on patient with biparietal osteodystrophy. J Korean Neurosurg Soc.

[4] Lara DA, Loar RW, Allen HD (2017). Visual diagnosis: baby with a scalp lesion, rash, and left-foot deformity. Pediatr Rev.

[5] Tsukada A, Yanaka K, Takeda H, Onuma K, Takada M, Nakamura K, Ishikawa E (2022). Idiopathic focal calvarial thinning: a case report. Surg Neurol Int.

[6] Yiu Luk S, Fai Shum JS, Wai Chan JK, San Khoo JL (2010). Bilateral thinning of the parietal bones: a case report and review of radiological features. Pan Afr Med J.

[7] Lenchik L, Sartoris DJ (1998). Orthopedic aspects of metabolic bone disease. Orthop Clin North Am.

[8] Kulak CA, Dempster DW (2010). Bone histomorphometry: a concise review for endocrinologists and clinicians. Arq Bras Endocrinol Metabol.

